# Integrin-αvβ3 as a Therapeutic Target in Glioblastoma: Back to the Future?

**DOI:** 10.3390/pharmaceutics14051053

**Published:** 2022-05-13

**Authors:** William Echavidre, Vincent Picco, Marc Faraggi, Christopher Montemagno

**Affiliations:** 1 Département de Biologie Médicale, Centre Scientifique de Monaco, 98000 Monaco, Monaco; wechavidre@centrescientifique.mc (W.E.); cmontemagno@centrescientifique.mc (C.M.); 2 Nuclear Medicine Department, Centre Hospitalier Princesse Grace, 98000 Monaco, Monaco; marc.faraggi@chpg.mc; 3 Institute for Research on Cancer and Aging of Nice, Centre Antoine Lacassagne, CNRS UMR 7284, INSERM U1081, Université Cote d’Azur, 06200 Nice, France

**Keywords:** glioblastoma, integrins, cilengitide, nuclear medicine, theranostics

## Abstract

Glioblastoma (GBM), the most common primary malignant brain tumor, is associated with a dismal prognosis. Standard therapies including maximal surgical resection, radiotherapy, and temozolomide chemotherapy remain poorly efficient. Improving GBM treatment modalities is, therefore, a paramount challenge for researchers and clinicians. GBMs exhibit the hallmark feature of aggressive invasion into the surrounding tissue. Among cell surface receptors involved in this process, members of the integrin family are known to be key actors of GBM invasion. Upregulation of integrins was reported in both tumor and stromal cells, making them a suitable target for innovative therapies targeting integrins in GBM patients, as their impairment disrupts tumor cell proliferation and invasive capacities. Among them, integrin-αvβ3 expression correlates with high-grade GBM. Driven by a plethora of preclinical biological studies, antagonists of αvβ3 rapidly became attractive therapeutic candidates to impair GBM tumorigenesis. In this perspective, the advent of nuclear medicine is currently one of the greatest components of the theranostic concept in both preclinical and clinical research fields. In this review, we provided an overview of αvβ3 expression in GBM to emphasize the therapeutic agents developed. Advanced current and future developments in the theranostic field targeting αvβ3 are finally discussed.

## 1. Introduction

Glioblastoma (GBM) is the most common malignant primary brain tumor, representing approximately 50% of all gliomas and 16% of all brain tumors [1]. GBM is the deadliest tumor of the central nervous system (CNS), with a median survival of only 15 months after initial diagnosis [2,3]. Despite an increasing understanding of its pathophysiology, GBM remains an incurable disease, with one-year and 5-year survival rates respectively of 39.7% and 5.5% [4]. These survival rates stand in stark contrast to the high survival rates of other common cancers such as breast (90%), prostate (90%), or colon (72%) [5]. Newly-diagnosed-GBM therapeutic management requires a multidisciplinary approach. The standard of care includes surgical resection, followed by radiation therapy and temozolomide (TMZ) administration, an alkylating chemotherapy agent [6,7,8]. Unfortunately, despite this multimodality therapy, over 60% of GBM patients are meant to relapse within one year [9]. Management of recurrent GBM remains a paramount challenge, as the treatment options are limited. Considering the clear need to improve therapeutic strategies, substantial efforts have been made to understand GBM biology. The blood-brain-tumor barriers, the intra-, and inter-tumoral heterogeneity, and the intrinsic resistance to chemo- and radio-therapies are important barriers to the development of effective treatments [10,11,12]. Underlying molecular mechanisms of GBM resistance to therapy were recently reviewed [13]. Low survival rates of GBM are, at least in part, a consequence of the extensive invasion of the brain tissue. This infiltration is notably controlled by the interactions between cancer cells and the surrounding brain microenvironment [14]. In addition to invasive features, GBM exhibits marked tumor cell proliferation and exacerbated angiogenesis. In all these mechanisms, integrins play fundamental roles and have therefore become attractive candidate molecules for therapeutic intervention in GBM [15]. Integrins are heterodimeric transmembrane complexes consisting of two subunits, α and β, able to form at least 24 different heterodimers [16]. Among them, the pro-angiogenic αvβ3 was the first to be found abundantly expressed in high-grade brain tumors [17,18]. αvβ3 belongs to the integrin subtypes that recognize the tripeptide sequence Arg-Gly-Asp (RGD) found in many extracellular matrix (ECM) proteins, including fibronectin or vitronectin. A plethora of studies highlighted the role of αvβ3 in sustaining a high proliferative rate, migrative and invasive properties of GBM, as well as promoting angiogenesis [18,19,20,21]. Consequently, research in the field of integrins has investigated the therapeutic potential of antibodies or chemicals targeting integrin-αvβ3 in GBM [22]. Targeted therapies are an important part of modern treatment concepts. As a central component of patient care and personalized medicine, nuclear imaging appears as a valuable tool to enhance patient selection and predict the treatment response [23]. Moreover, the emergence and advent of an innovative approach in the field of nuclear medicine, called theranostics, which consists of a single drug used for both diagnosis and therapeutic purposes (but labeled with different isotopes), have opened new opportunities for cancer management [24]. Several agents targeting αvβ3 and dedicated to these applications have been developed and could offer new theranostic strategies for GBM patient management [25]. In this review, we first provided an overview of the different roles fulfilled by integrin-αvβ3 in GBM, as well as different therapeutic strategies developed to target integrin-αvβ3. We finally emphasized the theranostic-dedicated αvβ3-targeting agents currently in development.

## 2. Role of Integrin-αvβ3 in GBM Progression

### 2.1. Integrin Signaling

Over 30 years ago, Takmun et al. were the first to propose “integrin” as a name for a protein complex linking the cell cytoskeleton to the ECM [26]. Integrins are a large family of transmembrane adhesion receptors, composed of non-covalent heterodimeric complexes involving α and β chains. Upon binding to ligands or ECM, integrins activate downstream signaling pathways which regulate a multitude of cellular effects in physiological and pathological situations. Once engaged with the ECM, integrins cluster and recruit various adaptor and signaling proteins in order to form focal adhesion complexes [27]. Focal adhesion kinase (FAK), which is activated by integrin-mediated ECM-adhesion, coordinates integrin signaling and promotes cell migration [28,29]. In addition, FAK expression was found to be correlated to increased invasiveness and recurrence in GBM [30,31,32]. These complexes activate intracellular downstream pathways including phosphoinositide-3-kinase (PI3K)/AKT, Src, or Ras mitogen-activated protein kinase (MAPK) pathways [33,34]. Cellular effects of integrin-activation result in cytoskeleton changes and lead to the activation of genes involved in proliferation, invasion, and survival [35]. While integrin-encoding genes are rarely mutated in neoplasms, deregulations of integrin signaling are frequent in GBM. Comparative immunohistochemistry staining of integrins in GBM reveals overexpression of α2, α3, α4, α5, α6, and β1, as well as αvβ3 and αvβ5 [21,34,36,37]. Integrins-αvβ3 and αvβ5 were the first identified as potential targets in GBM due to their involvement in several hallmarks of cancers. Accumulating evidence has demonstrated the pro-tumorigenic and pro-angiogenic role of αvβ3/αvβ5 in GBM, making them suitable targets for anti-cancer therapies. Data acquired from clinical samples revealed a prominent expression of αvβ3 by tumor and endothelial cells in the periphery of high-grade gliomas than αvβ5 [19]. Studies carried out in GBM demonstrated that αvβ3 expression is associated with poor prognosis and reduced time-to-progression [38]. This review therefore only focuses on αvβ3.

### 2.2. Expression of Integrin-αvβ3 in GBM

Integrin-αvβ3 was initially reported as the most important integrin in tumor angiogenesis [39]. Integrin-αvβ3 expression was also found to arise from glial cells, promoting proliferation, migration, and invasion of GBM. Integrin-αvβ3 and its ligand, vitronectin, are upregulated during the transition from low-grade tumors to advanced GBM [18,40,41]. Consequently, their expression was found to be associated with poor prognosis [17,42]. In the literature, integrin-αvβ3 expression has been evidenced by immunochemistry analyses in nearly 60% of GBM samples, whereas it is not expressed in normal brain tissue [43]. Additional studies demonstrated that integrin-αvβ3 expression was also correlated with poorer GBM prognosis [38]. Historically, αvβ3 expression was reported to be limited to angiogenic endothelial cells in the tumors, which suggests its involvement in GBM angiogenesis [18]. However, studies carried out on GBM-derived patient samples demonstrated that αvβ3 expression predominantly arises from glial cells rather than endothelial ones, as 85% of αvβ3 expression came from tumor cells themselves [17]. Taken together, these observations led to extensive investigations of αvβ3 roles in GBM progression.

### 2.3. Roles of Integrin-αvβ3 in Angiogenesis

Angiogenesis, the formation of new blood vessels, is a crucial process for the tumor to grow beyond 1–2 mm. Different integrins, including αvβ3, were found to promote angiogenesis by regulation of both proliferation and migration of endothelial cells [44]. Integrin-αvβ3 levels are relatively low in quiescent endothelium, whereas newly formed endothelial cells exhibit high levels of this integrin [45,46]. Consequently, to this observation, integrin-αvβ3 has garnered therapeutic attention for angiogenesis-dependant neoplasms. In 1994, Brooks et al. were the first ones to demonstrate that integrin-αvβ3 is a marker of angiogenic vasculature that can be targeted in oncology [47]. In a model of chick chorioallantoic membrane, the intravascular injection of integrin-αvβ3 antagonists, either cyclic peptides or monoclonal antibodies, was shown to disrupt ongoing angiogenesis by inducing apoptosis of vascular cells without affecting quiescent blood vessels [47]. Such anti-angiogenic effects of cyclic peptides were thereafter confirmed in 3D cultures of endothelial cells [48]. Mechanistically, integrin-αvβ3 was found to synergistically interact with VEGF to activate the VEGFR2 receptor, therefore promoting angiogenesis [49]. Recent work carried out with a three-dimensional, microfluidic angiogenesis model with immunosuppressive conditions, a key feature of GBM, demonstrated that αvβ3 interacts with M2-macrophages to drive blood vessel growth [50]. M2-macrophages are considered pro-tumoral and the M2 phenotype is driven by several stimuli including transforming growth factor-β (TGF-β). Dual inhibition of αvβ3 and TGF-β receptors was found to enhance the efficacy of anti-angiogenic treatment in GBM [50].

Considering that the pro-angiogenic activity of integrin-αvβ3 was initially reported as its main function, original-intended targets of αvβ3 antagonists were endothelial cells. Thus, the majority of αvβ3-targeting agents aimed to impair angiogenesis in solid tumors, including GBM. Nonetheless, preclinical studies demonstrated that integrin-αvβ3 plays a crucial role in the early steps of tumor angiogenesis, whereas its expression on endothelial cells is dispensable once tumor vasculature is established [51]. Over time, alternative means to maintain angiogenesis seem to be used instead of integrin-αvβ3-downstream signaling. These findings imply that the timing and duration of integrin-αvβ3 inhibition are critical factors that need to be considered when anti-integrin-αvβ3 agents administration is planned to aim at angiogenesis impairment.

### 2.4. Roles of Integrin in Migration and Invasion Processes

Whereas αvβ3 was restrictively considered for a long time as a pro-angiogenic factor, immunohistochemical analyses of GBM samples demonstrated that αvβ3 is more expressed by tumor cells rather than angiogenic ones [34]. These observations strongly suggest that αvβ3 plays a role in tumorigenesis, in addition to angiogenesis. More precisely, αvβ3 is preferentially expressed at the invasion front of GBM. In the complex regulatory network of tumor progression, integrins, as the main link between a cell and ECM, play an essential role in tumor invasion [52,53,54]. Integrin-αvβ3 supports cell adhesion to ECM through fibronectin, which enables the formation of tractions for migrating cells. Gene expression analyses of ECM components demonstrated an increased expression of fibronectin in GBM, as compared to the normal brain or non-invasive astrocytomas [41,55,56]. Additional data carried out in GBM biopsies showed that fibronectin and vitronectin promote local invasion of glioma cells [57,58]. Functional analyses revealed that fibronectin also promotes proliferation and resistance to irradiation [41]. In addition to fibronectin, integrin-αvβ3 expression was found to be associated with the invasive phenotype of glioma cells and to colocalize with matrix metalloproteinase 2 (MMP-2) [17,19]. MMPs are endopeptidases involved in tissue remodeling by proteolytic degradation of numerous ECM proteins, in physiological and pathological ways. Although multiple non-malignant cells, such as endothelial cells or macrophages, can secrete different isoforms of these proteases, glioma tissue has been shown to be one of the main sources of production of MMPs (mostly MMP-2 and MMP-9), as compared to normal brain tissue and other CNS tumors [59,60]. In this process, αvβ3-binding of MMP2 facilitates angiogenesis of tumor—invading endothelial cells, therefore promoting tumor growth [61]. Close interactions between the cellular matrix and GBM cells are fundamental for extensive tumor infiltration into neural tissue. Moreover, invasive properties of GMB have also been shown to rely on the requirement of αvβ3 and its downstream activator p21(RAC1)-activated kinase 4 (PAK4), as opposition forces to oncogene-induced senescence [62].

### 2.5. Integrin-αvβ3, Stemness and Drug Resistance

Advanced knowledge in GBM biology elicited mechanisms of its resistance to conventional therapies, including chemo- and radiotherapy [63,64]. In addition to the intrinsic resistance of cancer cells, their high heterogeneity, and the poor drug penetration through the blood-brain barrier, the tumor microenvironment was recently found to significantly impact the response to standard therapies. High levels of vitronectin and fibronectin were detected in clinical GBM tumors and found to confer cell-adhesion-mediated drug resistance [65]. In this setting, fibronectin suppressed p53-mediated apoptosis and upregulated P-glycoprotein expression, also known as multidrug resistance protein 1 (MDR1), making glioma cells chemoresistant.

In addition, increasing evidence has indicated the existence of a key population with stem cell properties, the glioma stem-like cells (GSCs), that are involved in resistance to chemotherapy and recurrence. Indeed, cancer stemness, referring to the stemcell-like phenotype of cancer cells, has been widely recognized as a vital player in tumorigenesis, from primary tumor development to disease relapse and treatment failure [66]. Studies on GSCs have highlighted the importance of paracrine signaling networks within the microenvironment in the maintenance of GSCs [67]. Indeed, GSCs interactions with the surrounding tumor microenvironment, notably with integrins and their ligands, were demonstrated to be a key driver of glioma progression, making tumor-microenvironment interactions a promising therapeutic target [68,69]. Consistently, several lines of evidence highlighted the enhanced propagation of GSCs with tumor-associated macrophages (TAM) infiltration [70]. In this setting, the interaction between periostin and αvβ3 was found to recruit TAMs in vivo and support tumor growth [71]. In addition to such observation, integrin-αvβ3 expression promotes tumor initiation, self-renewal, and resistance to erlotinib. Pharmacological inhibition of αvβ3 signaling reversed stemness and erlotinib resistance [72]. The involvement of αvβ3 in cancer stemness was also evidenced in other solid tumors such as breast cancer [73].

In addition, accumulating evidence suggests the role of integrin-αvβ3 in resistance to conventional therapies. Indeed, irradiation up-regulated integrin-αvβ3 expression in radiotherapy-treated human glioma cells. These results are in line with an increase in GBM-cell invasive capacities [62]. Based on integrin-αvβ3 induction in radioresistant glioma cells, some inhibitors of integrin-αvβ3 signaling were evaluated as radiosensitization agents. Specific αvβ3-inhibition using Cilengitide, an RGD-derived competitive inhibitor, or an ILK inhibitor, was found to radiosensitize GBM [74,75]. Preclinical studies showed that αvβ3-inhibition sensitizes GBM to temozolomide treatment by suppressing the recombination repair mechanisms [76]. Potentiation of radiation mediated by integrin-αvβ3 targeting suggests possible clinical translation for glioma therapy. Moreover, preclinical data demonstrated that integrin-αvβ3 is induced under hypoxia, a well-known cause of failure of radiotherapy in GBM [77]. Together, all these data suggest the involvement of αvβ3 in drug resistance and make it an attractive target for anti-cancer therapies.

## 3. Anti-Integrin-αvβ3 Agents for Anti-Cancer Therapy

In recent years, great progress has been made toward targeting integrins in cancer. Considering their particular involvement in GBM progression and chemo/radio-resistance, integrins are of great interest for targeted therapies, drug delivery, and tumor imaging. Several strategies have therefore been investigated to target αvβ3-overexpressing tumors, including antibodies or small molecules.

### 3.1. RGD-Derived Antagonists

Since its discovery, the core integrin-binding domain RGD in fibronectin attracted a lot of attention in the field of anti-cancer therapies. A variety of RGD-containing peptides were therefore developed to impair angiogenesis and tumorigenesis. Among these, a cyclic pentapeptide blocking the RGD binding site, cilengitide (cyclo-Arg-Gly-Asp-DPhe-NMe-Val), was identified as a selective αvβ3 and αvβ5 inhibitor. In vitro evaluations carried out on glioma cells showed that cilengitide induced their detachment and their death in a dose-dependent manner [78,79]. In preclinical models, cilengitide was found to impair GBM tumorigenesis through a multimodal action: anti-tumoral effects of cilengitide were mediated by anti-angiogenic, cytotoxic, and anti-invasive activities [80]. This agent was subsequently evaluated in clinical studies. In phase I/IIa study conducted on 52 patients with newly diagnosed GBM, cilengitide addition to standard chemoradiotherapy demonstrated moderate anti-tumor effects without toxicity [81]. Another phase II study conducted on 112 newly diagnosed GBM patients demonstrated an improved overall survival by comparison to the EORTC trial [82]. Instead, cilengitide addition to radiotherapy and temozolomide in a randomized phase III CENTRIC and phase II CORE trial did not show significant effects on overall survival (OS) of newly diagnosed GBM. Nonetheless, in the CORE study, higher αvβ3 levels in tumor cells were associated with improved OS in patients treated with cilengitide [83]. Reasons for such disappointing results include the high heterogeneity in αvβ3 levels, the unfavorable drug pharmacokinetics, and its dose-dependent opposing effects [84]. Indeed, whereas low doses were reported to stimulate angiogenesis, high doses appeared to impair it [85]. Despite these results, integrin inhibition remains a valuable anti-cancer strategy and several compounds showed promising preclinical. GLPG0187, a small integrin antagonist, showed effectiveness against glioma cells, as well as a safety profile in a phase I study conducted on 20 patients with progressive GBM [86,87]. Such compound remains to be further evaluated in advanced clinical phases, in combination with standard radio- or chemotherapies.

### 3.2. Integrin-αvβ3 and Drug Delivery

Considering its inherent safety and its ability to target both tumor and endothelial cells, RGD peptide can be used as a selective carrier to efficiently deliver anti-cancer drugs. Several examples of RGD peptide drug conjugates were proposed as targeted drug delivery systems [88,89,90]. Zhan et al. were among the first to design and evaluate paclitaxel (PTX)-loaded cyclo-RGD in preclinical studies. This combination approach significantly enhanced PTX anti-tumor effects, both in vitro and in vivo in the orthotopic GBM model [91]. Similar approaches using methotrexate or doxorubicin (DOX) displayed strong anti-glioma efficiency, representing a promising platform for therapy [92,93]. Integrin-*α*v*β*3 was more recently used as a target for the delivery of drug combinations. DOX- and PTX-loaded RGD-decorated micelles showed higher anti-glioma properties in comparison to DOX + PTX alone in intracranial U87-MG glioma-tumor-bearing mice [94]. Another chemotherapeutic agent, epirubicin, was also loaded into a cyclo-RGD-coated micelle and evaluated in a preclinical glioma model [95]. In vivo, this complex inhibited glioma growth by delivering high levels of epirubicin within the tumor. Despite their anti-tumor potential highlighted by preclinical studies, the use of RGD as a platform for drug delivery remains to be translated in the clinic.

### 3.3. Theranostics Development of αvβ3-Targeting Agents

**Figure 1:**
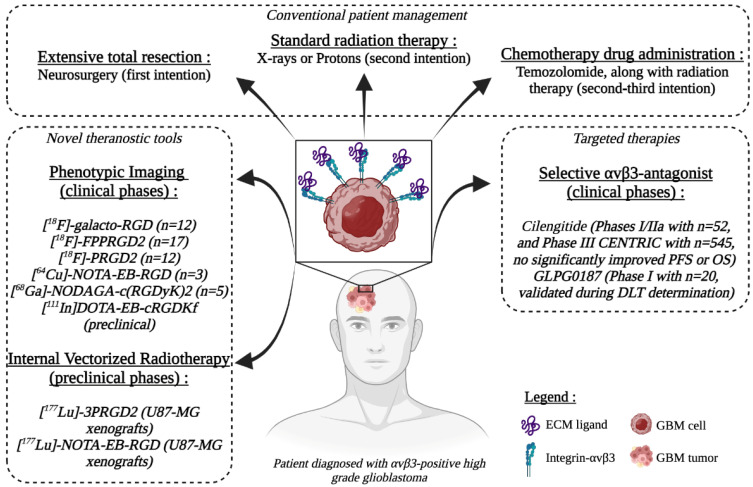
This panel depicts the standards of care for patients diagnosed with glioblastoma, from neurosurgery to radiation therapy, combined or not with chemotherapy. Current innovative tools in clinical phases for theranostics are listed. This illustration was created with BioRender (BioRender.com, accessed on 1 April 2022).

Nuclear medicine imaging is mainly based on the principle of using specific molecules (antibodies, chemicals...) labeled with radioactive isotopes to assess pathophysiological features of neoplasms. In addition to its potential to image a tumor antigen, nuclear medicine’s therapeutic potential can provide new opportunities for the management of GBM patients. In this way, the advent of theranostics has opened new avenues in the field of personalized treatments. The theranostic approach couples diagnostic imaging and therapy using the same molecule, or at least very similar, which is radiolabeled differently or administrated in different dosages. RGD-derived compounds dedicated to phenotypic imaging and radiation therapy are presented in Figure 1.

#### 3.3.1. Integrin-αvβ3 Targeting for Phenotypic Imaging

Non-invasive molecular imaging would be a precious tool to confirm the existence of the target and track its expression for monitoring tumor response. Since integrin-αvβ3 is an essential hallmark of tumor growth, invasion, and metastasis, imaging its expression is potentially interesting for patient risk stratification and patient selection for αvβ3-directed therapy. Several αvβ3-targeting radiotracers have been developed over the past decades and have been investigated for clinical translation. The majority of them are based on the tripeptide RGD because of its high affinity and specificity for integrin-αvβ3. This review only focuses on clinically available RGD-based tracers. All the clinically investigated RGD peptides displayed very similar in vivo distribution with high uptake in the urinary tract, due to urinary elimination of the tracer, and moderate liver and intestine uptakes. Additionally, no physiological brain uptake was found using different RGD peptides, making them putative tracers for GBM. [^18^F]-galacto-RGD was the first RGD tracer dedicated to positron emission tomography (PET) evaluated in humans. This compound was designed by conjugating a sugar amino acid to the cyclic peptide c(RGDfK). [^18^F]-galacto-RGD was evaluated for its ability to detect malignant lesions. In 12 GBM patients, [^18^F]-galacto-RGD demonstrated high heterogeneity in tumor uptake [96]. [^18^F]-galacto-RGD uptake was found to significantly correlate with αvβ3 expression assessed after immunohistological examinations. A significant correlation was observed regarding αvβ3 expression on tumor cells, whereas none was observed with microvessel-associated αvβ3 expression. This result provides further evidence that [^18^F]-galacto-RGD detects αvβ3-expressing tumor cells. To further improve affinity and tumor retention of RGD peptides, dimeric RGD peptides were developed and evaluated in several tumors, including GBM. [^18^F]-FPPRGD2 was the first one applied in clinical trials. Despite the low number of patients included in the study (n=17), [^18^F]-FPPRGD2 demonstrated higher detection rate of recurrent GBM in comparison to gold standard MRI imaging (100% vs. 93.3%) or [^18^F]-FDG (100% vs. 86.7%) [97]. Another RGD-peptide, the [18F]-PRGD2, was evaluated in 12 GBM patients. Unlike [^18^F]-FDG, [^18^F]-PRGD2 levels were correlated to the grade of GBM [98]. This result suggests that [^18^F]-PRGD2 is a potentially useful tool for assessing tumor grading. More recently, a first in humans trial using [^64^Cu]-NOTA-EB-RGD was conducted on 3 GBM patients. The introduction of the Evans Blue (EB), a dye molecule conjugated to an RGD-like agent harboring a metal chelator that can reversibly bind to circulating albumin, significantly enhanced tumor accumulation of integrin-αvβ3 radioligands. This was evidenced by the high tumor-to-background contrast over time of [^64^Cu]-NOTA-EB-RGD in recurrent GBM [99]. Nevertheless, this study should be conducted on a larger number of patients to confirm its potential.

Over the past several years, the use of gallium-68 (^68^Ga) for the development of imaging agents has considerably increased. Recent studies showed remarkable imaging potential of [^68^Ga]-RGD peptides in preclinical and early clinical phases. The potential of a ^68^Ga-radiolabeled dimeric RGD, the [^68^Ga]-NODAGA-c(RGDyK)_2_, was investigated in mice bearing U87-MG xenografts. Distribution in αvβ3-expressing tissues was significantly improved when using ^68^Ga-radiolabeled RGD compounds as compared to ^18^F-radiolabeled ones [100]. These results highlight the interest in ^68^Ga-radiolabeled RGD for imaging of αvβ3-expressing GBM. Another novel long-circulation integrin-targeted molecule, [^111^In]DOTA-EB-cRGDfK, showed significant tumor accumulation with high tumor-to-background ratio at 24h post-injection in U87-MG xenografts [101]. These radiotracers deserve further clinical investigations to confirm their potential. Their ability to specifically bind to αvβ3-expressing lesions holds the potential for appropriate patient selection, especially for those eligible to receive targeted-αvβ3 treatment.

#### 3.3.2. Integrin-αvβ3-Targeted Radiotherapy

The limited efficacy of cilengitide suggests the importance of integrin-αvβ3 imaging prior to treatment, but also to find an alternative to pharmacological inhibition. Although molecules that target αvβ3 have an acceptable safety profile, the interest in using them to tackle cancer has waned due to the lack of efficacy. Several factors, such as redundancy or compensatory mechanisms, could explain their limited efficacy in the clinic. The poor results of integrin pharmacological inhibition reported in GBM patients suggest the interest in finding new alternative approaches to target αvβ3. Indeed, the remarkable advances in nuclear medicine, especially in the field of targeted therapies, can provide new tools to treat GBM. Most therapeutic radiopharmaceuticals are labeled with beta-emitting isotopes (β-). These particles have a tissue penetration of only a few millimeters, which allows cell irradiation in limited radium, causing a cytotoxic effect on tumor cells while sparing surrounding healthy tissue. Commonly used β- emitters in clinical practice include lutetium-177 (^177^Lu, maximum tissue penetration of 2 mm) and yttrium-90 (^90^Y, maximum tissue penetration of 11 mm). ^177^Lu, with a half-life of 6.7 days and relatively high β- emission (Emax: 0.497 MeV), offers the advantage to deliver a high dose and achieving longer tumor radiation. Additionally, a unique feature of radionuclides is that they can exert the “cross-fire” effect, potentially destroying adjacent tumor cells. Most RGD peptides dedicated to internal vectorized radiotherapy of GBM have been evaluated in preclinical models using ^177^Lu-radiolabeled RGD peptides. [^177^Lu]-3PRGD2 was evaluated in mice models bearing U87-MG tumor (GBM) for its ability to impair tumorigenesis. U87-MG tumor growth was significantly delayed by a single intravenous injection of [^177^Lu]-3PRGD2 (5.55 GBq/kg; 111.0 MBq/0.2 mL; peptide 1.5 μg) [102]. Among the most promising RGD-peptides dedicated to internalized radiation therapy, [^177^Lu]-NOTA-EB-RGD was recently evaluated in patient-derived xenografts (PDXs) derived from non-small cell lung cancer [103]. [^177^Lu]-NOTA-EB-RGD was found to be highly retained into αvβ3-positive PDXs from 1 to 72 h post-injection. One unique injection of [^177^Lu]-NOTA-EB-RGD (18.5 MBq) completely eradicated tumor growth of αvβ3-positive PDXs. However, such compounds remain to be validated in GBM models. These preclinical data suggest that [^177^Lu]-NOTA-EB-RGD could be an efficient treatment option, that should be further evaluated in clinical trials.

## 4. Conclusions

Integrins are key actors of GBM progression and preclinical evidence suggests that their inhibition is an attractive and promising approach for treatment. Among them, integrin-αvβ3 was for a long time the most studied integrin due to its overexpression in GBM and its pro-tumorigenic role. Nevertheless, clinical studies aiming at targeting its downstream signaling pathways were restricted to tumors with high levels of αvβ3. The redundancy of integrins signaling and the lack of biomarkers greatly influence the response to targeted therapies involving small inhibitors, such as cilengitide. Improving patient selection, reducing side effects, and enhancing therapeutic efficacy are challenges in modern oncology. The recent advent of the theranostic approach in nuclear medicine could overcome this limitation. Vectorized nuclear medicine allows non-invasive molecular tumor characterization and personalized treatment. The great variability of αvβ3-expression and therefore the lack of patient stratification could somehow explain contradictory results obtained in clinical studies aiming at investigating the relevance of its inhibition in GBM. Reconsidering αvβ3-targeting agents would require accurate biomarkers to identify potential responding patients. Indeed, the efficacy of αvβ3-directed therapies is depending on the intra-tumor extent and integrin-αvβ3 distribution. Therefore, it is essential to assess αvβ3 expression to evaluate the eligibility of a GBM patient for targeted therapies. Several radiotracers dedicated to imaging have been developed and could be valuable tools to select eligible patients for such therapy. Finally, the therapeutic potential of nuclear medicine and its successful application to a broad range of tumors can be an additional therapeutic option for GBM. Despite the need for further clinical investigation for the majority of these compounds, they could have great potential to be integrated into combined therapies.

## Data Availability

Not applicable.
